# Conservative Management of Large Rectosigmoid Perforation under Peritoneal Reflection: Case Report and Review of the Literature

**DOI:** 10.1155/2015/364576

**Published:** 2015-03-31

**Authors:** G. G. Akgul, E. Yenidogan, Z. Ozsoy, I. Okan, H. A. Kayaoglu, S. Tali, M. Sahin

**Affiliations:** Department of General Surgery, Gaziosmanpasa University, Faculty of Medicine, 60150 Tokat, Turkey

## Abstract

Colonoscopy is accepted as the best method in diagnosis, treatment, and follow-up of colorectal diseases. As the amount of the usage of diagnostic and therapeutic colonoscopy rises, iatrogenic complications are more likely to be seen. The most important complications are perforations and bleeding. Whereas perforation is a complication that is seen rarely, because of the high ratio of morbidity and mortality, it should be analyzed more carefully. There is not a common view on the optimal treatment of colonoscopic perforation. Most cases require urgent surgery, and in some cases the iatrogenic perforation of colon can be managed by conservative methods. In this report, we present a rectosigmoid perforation under peritoneal reflection and conservative management of this case.

## 1. Introduction

Since colonoscopy was introduced in 1969 at the Department of Surgery of Beth Israel Medical Center in New York City, it is accepted as the best method in diagnosis, treatment, and follow-up of colorectal diseases [1]. As the amount of the usage of diagnostic and therapeutic colonoscopy rises, especially as a depending of the usage of aggressive therapeutic interventions such as polypectomy, iatrogenic complications are more likely to be seen.

Moderate and self-limited abdominal and anal pain and flatulence and diarrhea are the most commonly seen complications. The other less commonly seen complications are pneumothorax, pneumoperitoneum, mesenteric tears, volvulus, incarcerations of hernia, and retroperitoneal abscess [2]. The most important complications are perforations and bleeding. Whereas perforation is a complication that is seen rarely, because of the high ratio of morbidity and mortality, it should be analyzed more carefully. Several large, retrospective studies have determined perforation incidences of 0.02–0.8% and 0.15–3% for diagnostic and therapeutic colonoscopy, respectively [3].

There is not a common view on the optimal treatment of colonoscopic perforation. The traditional management of iatrogenic perforation is surgical repair by either laparotomy or laparoscopy. However, most cases require urgent surgery, and in some cases the iatrogenic perforation of colon can be managed by conservative methods. In this report, we present a rectosigmoid perforation under peritoneal reflection and conservative management of this case.

## 2. Case Presentation

A 78-year-old woman presented with a 6-month history of abdominal discomfort like pain, distention. The physical examination was unremarkable. All other laboratory test results were within normal ranges. The patient did not have significant past medical or surgical history and our patient had never had a previous screening study for colorectal cancer. Thus, we decided to perform colonoscopy. Colonoscopic examination was performed with the patient under conscious sedation with intravenous 2 mg midazolam and 50 mg pethidine.

**Figure 1:**
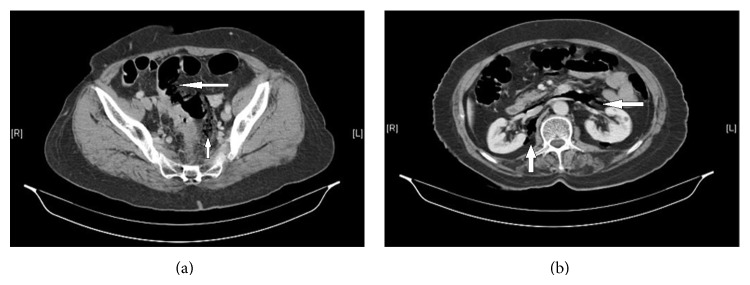
(a) Rectosigmoid perforation under peritoneal reflection and free air in retroperitoneal space. (b) Para-aortic and pararenal free air.

During insertion and withdrawal, the colonoscopy revealed no pathologic findings. However the patient felt uncomfortable and complained of severe pain. An approximately 5 cm perforation was observed 15 cm above the anal verge, located at upper posterior rectum and rectosigmoid conjunction during the withdrawal of the colonoscopy. Abdominal examination demonstrated a soft abdomen, with a mild suprapubic and left lower tenderness, no distension and without peritoneal sign. Initial upright plain abdominal and chest X-ray films were normal; no free air was seen. The patient then underwent urgent contrasted computed tomography of the abdomen and revealed large amount of air in the retroperitoneal space but no signs of peritoneal perforation were detected (Figures 1(a) and 1(b)).

Conservative management was administered, oral intake was stopped, and intravenous antibiotics were given. In the following days, the patient's vital signs were stable. Patient had had nothing oral for 4 days. After 4 days, liquid diet had been started because there was not any sign of peritonitis. The patient was discharged on the 10th day. In the first month of following up the patient, all the vital findings were observed as stable.

## 3. Discussion

Although colonic perforation is rarely seen during colonoscopy, it is still the most important complication of all. Besides, the consensus on the treatment of perforation has not been ensured. There are some cases that should be considered for the method of approach to colonoscopic perforations: the mechanism of perforation, the size of perforation, adequate bowel preparation, elapsed time of diagnosis, and the clinical condition of patient and primer colonic pathology.

Three main mechanisms of colonic perforations are [4] (1) mechanical perforation by the endoscope's tip or loop (often in a difficult bend), (2) barotraumas from hyperinsufflation that generally affects the caecum, and (3) therapeutic procedures such as electrocoagulation for polypectomy and laser or organ plasma coagulation (generally small and diagnosed rarely during the procedure).

Mechanical perforations (traction) are generally larger and more related to morbidity and mortality. Thus, they are needed to be approached more aggressively [5]. The perforations that occur during therapeutic treatment are generally small and diagnosed rarely during the procedure. Because of the small-sized perforations, peritoneal contamination can be seen less. General condition of the patient is more regular and in this group conservative approach is usual [6].

The most challenging areas in scope transition are sigmoid colon, rectosigmoid junction, and hepatic flexure [7]. In perforations that developed in distal rectosigmoid junction, air is generally seen in the retroperitoneal area. With this perspective, colonoscopic perforations are divided into two groups: first one is intraperitoneal perforations and second one is extraperitoneal perforations. Extraperitoneal perforations are usually seen at the below rectum and are retroperitoneal. Because of this, abdominal and pelvic CT should be used to set the location of the perforation, to expose abscess and intra-abdominal liquid and peritoneal and retroperitoneal air [8]. The approaches of treatment options are conservative, endoscopic, and operative management (open or laparoscopic approach). Every treatment should be personalized according to patient's clinical condition. If perforation is suspected during colonoscopy, the patient should be followed carefully. Early diagnosis and treatment are the cornerstones to reduce morbidity and mortality.

Conservative approach is an option that should be considered in the group of patients whose general condition is stable and who do not have peritoneal irritation symptoms. Generally accepted conservative approach is to rest bowels by stopping oral diet, intravenous antibiotics, and hydration and following up in between 3 and 6 hours [9].

Pneumoperitoneum is not an indication for operative management by itself. Patients with pneumoperitoneum, who are well chosen and clinically stable and do not have peritoneal irritation symptoms, can also be treated by conservative approach. If the conservative treatment succeeds, the clinical appearance of patient gets better in 24–48 hours. Otherwise, if clinical improvement is not seen, complicated intra-abdominal infections should be considered and should be treated more aggressively. In various studies the conservative treatment of colonoscopic perforation succeeds in between 33 and 73% [10].

Recent retrospective studies show that the percentage of conservative approach has been increasing [11]. The advantages of conservative management on appropriate patients are the shortening of the duration of hospital stay, early return to daily life, and less morbidity [1, 6, 11]. Reports evaluating the conservative management and outcome of colonoscopic perforation are summarized in Table 1.

The interesting point in this case is that the perforation developed during diagnostic colonoscopy is treated by conservative approach, contrary to literature. Even though there is a perforation in the length of approximately 5 cm segment on this patient's rectosigmoid junction posterior wall, the patient has been treated with conservative approach without any complications. This reminds us of a question. Can the conservative management be the first option without considering the perforation mechanism of the perforations under peritoneal reflections?

## Figures and Tables

**Table 1:** Summaries of the reports evaluating the conservative management and outcome of colonoscopic perforation.

	Number of perforations	Conservative	Failed conservative	Morbidity conservative	Mortality conservative
Avgerinos et al. [1]	35	12 (34%)	1	0	0
Sagawa et al. [3]	8	4 (50%)	0	0	0
Lüning et al. [4]	35	1 (3%)	1	0	0
Castellví et al. [6]	54	12 (22%)	2	0	0
Iqbal et al. [11]	72	10 (14%)	0	1	1
Tran et al. [12]	21	2 (9.5%)	2	0	0
Korman et al. [13]	37	2 (5.5%)	0	0	0
Cobb et al. [14]	14	3 (21.5%)	2	0	0
Heldwein et al. [15]	26	12 (46.2%)	0	0	0
Tulchinsky et al. [16]	7	1 (14%)	0	0	0
Mai et al. [17]	23	1 (4%)	0	0	0
Coimbra et al. [18]	43	1 (2%)	0	0	0
García Martínez et al. [19]	15	3 (20%)	0	0	0
Tam and Abbas [20]	26	4 (15%)	0	1	0
Anderson et al. [21]	20	1 (5%)	0	0	1
Araghizadeh et al. [22]	31	11 (35)	3	1	1
Christie and Marrazzo [23]	7	5 (71.5%)	0	0	0
Farley et al. [24]	45	3 (6.5%)	0	0	0
Hall et al. [25]	15	1 (6.5)	0	0	0
